# Common DNA methylation alterations of Alzheimer's disease and aging in peripheral whole blood

**DOI:** 10.18632/oncotarget.7862

**Published:** 2016-03-02

**Authors:** Hongdong Li, Zheng Guo, You Guo, Mengyao Li, Haidan Yan, Jun Cheng, Chenguang Wang, Guini Hong

**Affiliations:** ^1^ Key Laboratory of Ministry of Education for Gastrointestinal Cancer, Department of Bioinformatics, Fujian Medical University, Fuzhou, China; ^2^ College of Bioinformatics Science and Technology, Harbin Medical University, Harbin, China; ^3^ Department of Preventive Medicine, School of Basic Medicine Sciences, Gannan Medical University, Ganzhou, China

**Keywords:** aging, Alzheimer's disease, DNA methylation, peripheral whole blood, Gerotarget

## Abstract

Alzheimer's disease (AD) is a common aging-related neurodegenerative illness. Recently, many studies have tried to identify AD- or aging-related DNA methylation (DNAm) biomarkers from peripheral whole blood (PWB). However, the origin of PWB biomarkers is still controversial. In this study, by analyzing 2565 DNAm profiles for PWB and brain tissue, we showed that aging-related DNAm CpGs (Age-CpGs) and AD-related DNAm CpGs (AD-CpGs) observable in PWB both mainly reflected DNAm alterations intrinsic in leukocyte subtypes rather than methylation differences introduced by the increased ratio of myeloid to lymphoid cells during aging or AD progression. The PWB Age-CpGs and AD-CpGs significantly overlapped 107 sites (*P*-value = 2.61×10^−12^) and 97 had significantly concordant methylation alterations in AD and aging (*P*-value < 2.2×10^−16^), which were significantly enriched in nervous system development, neuron differentiation and neurogenesis. More than 60.8% of these 97 concordant sites were found to be significantly correlated with age in normal peripheral CD4^+^ T cells and CD14^+^ monocytes as well as in four brain regions, and 44 sites were also significantly differentially methylated in different regions of AD brain tissue. Taken together, the PWB DNAm alterations related to both aging and AD could be exploited for identification of AD biomarkers.

## INTRODUCTION

Alzheimer's disease (AD) is the most common form of neurodegenerative illness. One important risk factor for its occurrence is aging [1, 2]. DNA methylation (DNAm) as an important epigenetic mechanism is closely associated with aging and AD progression [3]. For example, it has been reported that the decreased promoter methylation of *BACE* and *PS1* genes during aging in brain tissue could lead to the development of sporadic AD [4]. Therefore, the investigation of aging- and AD-related DNAm alterations and analysis of their relationship will help reveal the underlying pathogenesis of AD and provide clues for AD biomarker identification.

As peripheral whole blood (PWB) sampling is non-invasive and easy to handle, researchers have considered PWB as a promising surrogate for tissue to investigate disease associated molecular biomarkers [5-7]. Many DNAm alterations have been identified from PWB of elderly people and of people with aging-related neurodegenerative illnesses [5, 8-10]. Notably, as DNA in PWB is derived from a mixture of various leukocyte subtypes (mainly grouped into myeloid and lymphoid cells) with distinct DNA methylation patterns, both the proportion changes and intrinsic DNAm pattern alterations of leukocyte subtypes could influence the DNAm signals observed in PWB [11]. To clarify where these aberrant DNAm signals originate will help us understand the underlying mechanism of disease. Until now, however, there are no conclusions on the origin of aging-related DNAm alterations observed in PWB: some researchers believe that aging-related PWB DNAm alterations influenced by leukocyte cell proportion changes are limited [9], while other researchers consider that most of the aging-related PWB DNAm alterations could be explained by the varied cell compositions [12]. Actually, the shifts of cell proportions and altered DNAm alterations intrinsic in peripheral leukocytes have both been observed in PWB samples of elderly people, as well as in PWB of patients with AD [12-14]. Nevertheless, to the best of our knowledge, no study has focused on analysis of the origin of PWB DNAm alterations observed in patients with AD. On the other hand, many aging-related co-methylation modules identified from PWB, which mainly enriched in neuron differentiation and development, cell fate commitment and embryonic morphogenesis associated functional categories, have also been identified from brain tissue of elderly people [15]. However, the relationship between AD-related DNAm alterations observed in PWB and the DNAm alterations observed in brain tissue is still unclear, the specification of which will provide perspectives on disease pathogenesis and biomarker identification.

In this study, using multiple PWB DNAm profile data sets, we revealed that both aging-related DNAm CpGs (Age-CpGs) and AD-related DNAm CpGs (AD-CpGs) observable in PWB mainly reflect intrinsic DNAm alterations of leukocytes. Then, after removing those DNAm alterations which were likely affected by proportion shifts of leukocyte subtypes, we showed that Age-CpGs and AD-CpGs in PWB significantly overlapped with concordant alterations. These overlapped sites were observed to be significantly correlated with age in peripheral CD4^+^ T cells and CD14^+^ monocytes as well as in four regions of normal brain tissue and differentially methylated in brain tissues between AD and normal controls, suggesting that they could serve as candidate biomarkers in PWB for AD identification.

## RESULTS

### Genome-wide identification of Age-CpGs from PWB

PWB DNAm profiles used to identify Age-CpGs were determined from 647 normal individuals aged between 16 and 101 years from six data sets (Set 1 to 6, detailed information was described in Table 1). Using linear regression model with a false discovery rate (FDR) < 0.05, we identified 1270, 1490, 127, 267, 134 and 325 Age-CpGs from Set 1 to 6, respectively. Pairwise comparison of the six lists showed that the Age-CpGs identified from every two PWB data sets significantly overlapped (*P*-value < 2.2×10^−16^, hypergeometric test), and all overlapped Age-CpGs had significantly concordant positive or negative correlations with age (all *P*-values < 2.2×10^−16^, binomial test, Table 2). These results suggested that the Age-CpGs detected from the six PWB data sets were significantly reproducible. We integrated the six lists of PWB Age-CpGs into a list of 1807 Age-CpGs, hereafter referred to as PWB Age-CpGs, according to the criteria described in the Methods section.

**Table 1:** DNA methylation data sets analyzed in this study

Data set	Sample num^a^	Tissue^b^	Description	Gender (M:F)	Age (mean, yrs)	Platform	GEO accession num	Ref
Set 1	173 (20)	PWB	healthy Dutch cohorts	88:85	16~59 (29.8)	27K	GSE41037	[15]
Set 2	92 (13)	PWB	healthy Dutch cohorts	38:54	16~65 (38.8)	27K	GSE41037	[15]
Set 3	84 (12)	PWB	healthy Dutch cohorts	52:32	34~88 (63.4)	27K	GSE41037	[15]
Set 4	233 (41)	PWB	healthy United Kingdom women	0:233	52~78 (68.9)	27K	GSE19711	[25]
Set 5	74 (16)	PWB	healthy Caucasian cohorts	29:45	47~101 (73.8)	450K	GSE40279	[26]
Set 6	71 (13)	PWB	healthy Caucasian cohorts	48:23	28~86 (58.2)	450K	GSE40279	[26]
Set 7	46 (0)	leukocyte subtype	healthy cohorts	---	---	450K	GSE39981	[27]
Set 8	187 (27)	CD4^+^T cell	MESA cohorts	---	45-79 (58.1)	450K	GSE56581	[13]
Set 9	1011(91)	CD14^+^ monocyte	MESA cohorts	---	45~79 (58.1)	450K	GSE56046	[13]
Set 10	121 (15)	cerebellum	healthy Caucasian cohorts	76:30	16-96 (46.2)	27K	GSE15745	[28]
Set 11	133 (20)	frontal cortex	healthy Caucasian cohorts	77:36	16-101 (47.3)	27K	GSE15745	[28]
Set 12	125 (17)	pons	healthy Caucasian cohorts	77:31	15-101 (47.0)	27K	GSE15745	[28]
Set 13	127 (21)	temporal cortex	healthy Caucasian cohorts	69:37	15-101 (49.0)	27K	GSE15745	[28]
	AD:control							
Set 14	48:9	PWB	MRC London Brainbank cohorts	17:40	70-96 (83.2)	450K	GSE59685	[5]
Set 15	58:21	entorhinal cortex	MRC London Brainbank cohorts	31:48	65-96 (83.1)	450K	GSE59685	[5]
Set 16	60:24	frontal cortex	MRC London Brainbank cohorts	33:55	65-96 (83.1)	450K	GSE59685	[5]
Set 17	61:26	superior temporal gyrus	MRC London Brainbank cohorts	34:53	65-96 (82.9)	450K	GSE59685	[5]
Set 18	60:23	cerebellum	MRC London Brainbank cohorts	34:49	65-96 (82.7)	450K	GSE59685	[5]

a The number inside the parentheses indicates the number of removed samples and the number outside the parentheses indicates the number of samples analyzed in this study

b PWB: peripheral whole blood

c 27K: Illumina Infinium Human Methylation27 BeadChip; 450K: Illumina Infinium HumanMethylated450k BeadChip.

### PWB Age-CpGs mainly reflect Age-CpGs of leukocyte subtypes

To evaluate the origin of PWB Age-CpGs, we examined the contribution of the proportion changes and intrinsic DNAm alterations of leukocyte subtypes to PWB signals during aging, respectively. By employing the deconvolution algorithm [16], we estimated the proportions of each leukocyte subtype in PWB of healthy people sampled in Set 1 to 4. As leukocytes in PWB can be classified into two classes, the lymphoid and myeloid cells, and the inter-class differences of DNAm levels are larger than the intra-class differences [11], we evaluated the associations of proportions of myeloid and lymphoid cells with age using Spearman's rank correlation test, respectively. The results showed that, in all four data sets, the proportions of myeloid cells in PWB were significantly positively correlated with age (*r* = 0.14 ~ 0.20, all *P*-values < 0.05), while the proportions of lymphoid cells in PWB were significantly negatively correlated with age (*r* = −0.15 ~ −0.22, all *P*-values < 0.05).

Given that the ratio of myeloid cell proportion to lymphoid cell proportion in PWB increases during aging, if a PWB Age-CpG is mainly determined by the leukocyte cell proportion shifts, the correlation state (positive or negative correlation) between its DNAm level and age should depend on its relative DNAm level in myeloid cells compared to lymphoid cells [17]. Thus, if an Age-CpG was observed to be positively (or negatively) correlated with age in PWB and hypermethylated (or hypomethylated) in myeloid cells compared to lymphoid cells, we considered this PWB Age-CpG was concordant with the cell population shifts. In the following analysis, we used the concordance rate, defined as the percentage of concordant Age-CpGs among all PWB Age-CpGs, to evaluate the extent that the cell population shifts could contribute to the PWB Age-CpGs (see Methods).

**Figure 1:**
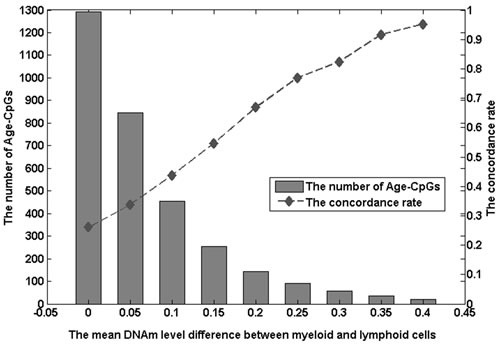
The number of Age-CpGs and the concordance rate of Age-CpGs under different mean methylation level differences between myeloid and lymphoid cells The mean difference of methylation levels between myeloid cells and lymphoid cells is plotted against the number of Age-CpGs (grey bars; left axis scale) or the concordance rate of Age-CpGs with ML-CpGs (dashed line; right axis scale). Age-CpGs are the age-related DNAm CpG sites in peripheral whole blood.

We identified differentially methylated CpG sites between myeloid and lymphoid cells from the DNA methylation profiles of purified PWB leukocyte subtypes (Set 7 described in Table 1) by using *t*-test. Totally, with an FDR < 0.05, 6817 CpGs, denoted as ML-CpGs, were found to be significantly differentially methylated between the two groups of leukocytes. Compared the PWB Age-CpGs with ML-CpGs, we found that 71.6% (1294) of the 1807 PWB Age-CpGs were included in the ML-CpGs, but the concordance rate was only 26% (*P*-value > 0.99, binomial test), indicating that about 74% of the overlapped Age-CpGs were unlikely to be determined by the proportion shifts of myeloid and lymphoid cells and may reflect the intrinsic DNAm alterations in leukocytes. However, we found that the concordance rate increased as the DNAm level differences between myeloid and lymphoid cells increased. As shown in Figure 1, when the mean DNAm level difference was larger than 0.35, the concordance rate of overlapped PWB Age-CpG was 91.3% (*P*-value < 1.36×10^−7^, binomial test). This result indicated that only part of PWB Age-CpGs were introduced by proportion shifts of leukocyte subtypes.

To examine the contribution of intrinsic DNAm alterations of leukocyte subtypes to PWB alterations observed during aging, DNAm profiles for CD4^+^ T cells (Set 8 described in Table 1) and CD14^+^ monocytes (Set 9 described in Table 1) were collected from the Multi-Ethnic study of Atherosclerosis (MESA, [13]). Using linear regression model, with an FDR < 0.05, we identified 809 and 3326 Age-CpGs for peripheral CD4^+^ T cells and CD14^+^ monocytes, respectively. We found that 386 of the 809 Age-CpGs observed in CD4^+^ T cells were included in the PWB Age-CpGs and 99.74% (385) of them had concordant positive or negative correlations with age in CD4^+^ T cells and PWB, which was unlikely to happen by chance (*P*-value < 2.2×10^−16^, binomial test). Similarly, 1046 of the 3326 Age-CpGs identified from CD14^+^ monocytes were included in the PWB Age-CpGs and 99.24% (1038) of them had significantly concordant positive or negative correlations with age in CD14^+^ monocytes and PWB (*P*-value < 2.2×10^−16^, binomial test).

These results indicated that the Age-CpGs observed in PWB may mainly reflect intrinsic Age-CpGs of leukocyte subtypes and partly reflect CpGs with large DNAm differences between myeloid and lymphoid cells easily affected by their proportion changes.

**Table 2:** Comparison of Age-CpGs respectively identified from six data sets

Data set	Set 1(1270)	Set 2(1490)	Set 3(127)	Set 4(267)	Set 5(134)	Set 6(325)
Set 1 (1270)^a^	---	747*(100%)	85*(100%)	174*(100%)	64*(100%)	153*(100%)
Set 2 (1490)	747*(100%)^b^	---	97*(100%)	191*(100%)	76*(100%)	181*(100%)
Set 3(127)	85*(100%)	97*(100%)	---	45*(100%)	40*(100%)	65*(100%)
Set 4(267)	174*(100%)	191*(100%)	45*(100%)	---	28*(100%)	71*(100%)
Set 5(134)	64*(100%)	76*(100%)	40*(100%)	28*(100%)	---	72*(100%)
Set 6(325)	153*(100%)	181*(100%)	60*(100%)	71*(100%)	72*(100%)	---

a The number inside the parentheses indicates the number of Age-CpGs identified from the data set indicated outside the parentheses

b The number outside the parentheses indicates the number of overlapping Age-CpGs and the percentage inside the parentheses indicates the proportion of concordant overlapping Age-CpGs identified from the data sets indicated in the corresponding row and column, respectively

* represent the FDR adjusted *P*-value < 0.05.

### AD-CpGs identified from PWB

**Figure 2:**
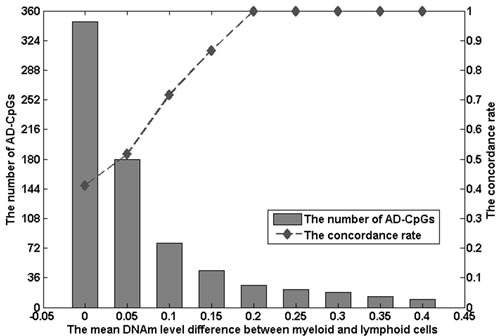
The number of AD-CpGs and the concordance rate of AD-CpGs under different mean methylation level differences between myeloid and lymphoid cells The mean difference of methylation levels between myeloid cells and lymphoid cells is plotted against the number of AD-CpGs (grey bars; left axis scale) or the concordance rate of AD-CpGs with ML-CpGs (dashed line; right axis scale). AD-CpGs are the Alzheimer's disease related DNAm CpG sites in peripheral whole blood.

PWB DNAm profiles used to identify AD-CpGs were determined from 57 individuals including 48 AD patients and 9 normal controls (Set 14 described in Table 1) collected from MRC London Neurodegenerative Disease Brain Bank [5]. Using the RankProd method [18] with an FDR < 0.05, we identified 805 significantly differentially methylated CpGs between AD patients and normal controls, denoted as PWB AD-CpGs. Similarly, as the ratio of the myeloid cell proportion to the lymphoid cell proportion tends to increase in AD PWB [14], if a PWB AD-CpG was observed to be hypermethylated (or hypomethylated) in AD PWB compared to normal controls and correspondingly observed to be hypermethylated (or hypomethylated) in myeloid cells compared to lymphoid cells, we considered this PWB AD-CpG was concordant with the cell population shifts. We also used the concordance rate to evaluate the extent that the cell population shifts could contribute to the PWB AD-CpGs. Compared the AD-CpGs with ML-CpGs, we found that 347 of the 805 AD-CpGs overlapped with the ML-CpGs and the concordance rate was 41.2%, indicating that more than a half of the PWB AD-CpGs were not determined by the cell proportion shifts. Notably, AD-CpGs with large DNAm level differences between myeloid and lymphoid cells also tended to be easily affected by the cell proportion changes. As shown in Figure 2, when the mean DNAm level difference was larger than 0.2, the concordance rate of the overlapped PWB AD-CpGs was 100%. According to these results, we considered that the AD-CpGs observed in PWB could reflect the CpGs with large DNAm difference between myeloid and lymphoid cells easily affected by leukocyte proportion changes during AD progression and may also mainly reflect intrinsic AD-CpGs of leukocyte subtypes.

### Common Age-CpGs and AD-CpGs in PWB

To evaluate the relationship between Age-CpGs and AD-CpGs observed in PWB, we first respectively removed those CpGs likely affected by the proportion changes of myeloid and lymphoid cells during aging and in AD progression. Finally, we obtained 1469 Age-CpGs and 662 AD-CpGs, respectively. We found that these two lists overlapped 107 sites, which was significantly more than what could be observed by random chance (*P*-value < 2.61×10^−12^, hypergeometric test). Among these 107 overlapped CpGs, 90.6% (97) were observed to be positively (or negatively) correlated with age in PWB and concordantly hypermethylated (or hypomethylated) in AD PWB samples compared to normal controls, significantly higher than what could be expected by chance (*P*-value < 2.61×10^−12^, binomial test). Furthermore, we also found that 59 of these 97 sites tended to be significantly positively (or negatively) correlated with age in both CD4^+^ T cells and CD14^+^ monocytes (Table S1), suggesting that the common Age-CpGs and AD-CpGs in PWB could have common alterations in these two types of leukocytes.

Functional enrichment analysis showed that genes mapping by these 97 CpGs were enriched in nervous associated biological progresses, including nervous system development, neuron differentiation and neurogenesis (Fisher's exact test, FDR < 0.05) (Table S2). This result suggested that aging-related DNAm alterations in these CpGs may play roles in promoting the progression of AD. In the following analysis, we denoted these 97 CpGs as PWB AD-Age-CpGs.

### PWB AD-Age-CpGs in brain tissue

To evaluate whether the PWB AD-Age-CpGs could be associated with aging in brain tissue, DNAm profiles for cerebellum, frontal cortex, pons and temporal cortex were obtained from 150 neurologically normal Caucasian subjects with no clinical history of neurological or cerebrovascular diseases as described in Set 10 to 13 in Table 1. Using linear regression model, with an FDR < 0.05, we identified 3490, 5455, 7206 and 8973 Age-CpGs from these four brain regions, respectively. Among the 97 AD-Age-CpGs, 60, 63 and 77 CpGs overlapped with Age-CpGs identified from frontal cortex, pons and temporal cortex (*P*-value < 2.2×10^−16^, hypergeometric test, Table S1), respectively, and more than 98.4% of them showed concordant positive or negative correlations with age in PWB and brain tissue. Although only 11 Age-CpGs observed in cerebellum overlapped with the 97 AD-Age-CpGs, 10 of them had concordant positive or negative correlations with age, which could not be observed by chance (*P*-value = 5.9×10^−3^, binomial test).

To further evaluate whether the PWB AD-Age-CpGs could be associated with AD in brain tissue, we collected four data sets of DNAm profiles of brain tissues of AD patients and normal controls sampled from cerebellum, entorhinal cortex, prefrontal cortex and superior temporal gyrus respectively (Set 15 to 18 described in Table 1), which were obtained from 117 individuals archived in the MRC London Neurodegenerative Disease Brain Bank. Using the RankProd method, with an FDR < 0.05, we found that 44 of the PWB AD-Age-CpGs were significantly differentially methylated in at least one of the four brain regions of AD compared to normal controls (9, 22, 22 and 22 AD-Age-CpGs for the four brain tissues respectively, Table S3).

These results indicated that common DNAm alterations observed in PWB Age-CpGs and AD-CpGs might not alter in a tissue-specific manner.

## DISCUSSION

Controversy in origin of PWB DNAm signals in patients with aging-related diseases arises from the observed increased ratio of myeloid to lymphoid cell proportions during disease progression [11]. Our analysis revealed that Age-CpGs observed in PWB mainly reflect intrinsic DNAm alterations of leukocytes and partly reflect those CpGs with large DNAm level differences between the myeloid cells and lymphoid cells. Although we were unable to further analyze the AD-CpGs in PWB leukocyte subtypes due to the lack of DNAm profiling data for leukocyte subtypes of AD patients, we believe that AD-CpGs observed in PWB also mainly reflect intrinsic DNAm alterations of leukocytes as the concordance rate between AD-CpGs and CpGs differentially methylated in myeloid cells compared to lymphoid cells was as low as 41.2%. Therefore, we suggested an improved approach to identify DNAm biomarkers for Alzheimer's disease and aging in PWB by focusing on those CpG sites with no or small DNAm level differences between myeloid cells and lymphoid cells.

Age-CpGs and AD-CpGs in PWB have shown significant overlaps with consistent changes: positively correlated with age and hypermethylated in AD or negatively correlated with age and hypomethylated in AD. Among the overlapped CpGs, we found many genes have been reported to be AD-associated genes. For example, by inhibiting the *ADAM10* metalloprotease, *Sfrp1* has been considered to play an important role in pathological events of Alzheimer's disease [19]. *DPYS*, *IGF1R*, *NRG1* and *GRB14* are collected in AlzGene database ([20], http://www.alzgene.org/) which provides a comprehensive field synopsis of genetic association studies performed in Alzheimer's disease. In our analysis, these five genes have been found hypermetylated during aging in PWB as well as in brain tissue. This phenomenon provides a new evidence that aging could be an important causal mechanism leading to AD progression.

Common DNAm alteration patterns have been found in PWB and brain tissue in this study by examining the DNAm changes of PWB Age-CpGs and AD-CpGs in normal brain tissues of elderly people and AD patients. Recently, such common DNAm alternations have become the focus of some aging-related DNAm alteration investigations involving PWB and solid tissue. In a previous study, researchers have reported 749 aging-related CpGs identified from PWB, which could also be identified in multiple solid tissue samples including human embryonic stem cells. They believe that these Age-CpGs could come from stem cells [21]. Among these 749 CpGs, 42 were included in the PWB AD-Age-CpGs. We also found that some of them were differentially methylated in different regions of brain tissue of AD patients and tended to significantly correlate with age in both CD4^+^ T cells and CD14^+^ monocytes. Therefore, we inferred that the common aging- and AD-related alterations observed in PWB may originate from stem cells [19, 20], which need further investigation and validation.

Though we have observed common DNAm alterations in PWB of elderly people and AD patients, we note that some specific DNAm patterns may exist in AD blood. Among the peripheral leukocytes, monocytes have been reported to be more proximal to the pathogenic cell type [22]. Therefore, we compared the AD-CpGs observed in PWB with the Age-CpGs observed in CD14^+^ monocytes. We found that, only 66.7% of the overlapped sites between PWB AD-CpGs and CD14^+^ monocyte Age-CpGs had consistent changes: positively (or negatively) correlated with age and correspondingly hypermethylated (or hypomethylated) in AD PWB samples. In contrast, the consistent rate for CD4^+^ T cell Age-CpGs and PWB AD-CpGs was 92.7%. This hinted us that specific DNAm alterations could exist during AD especially in monocytes [23], which may serve as genetic risk factors for AD and need further study.

## MATERIALS AND METHODS

### DNA methylation data

All data sets analyzed in this study were downloaded from the Gene Expression Omnibus (GEO) repository [24] (http://www.ncbi.nlm.nih.gov/geo/; Table 1). Set 1 to 6 were used for the detection and reproducibility evaluation of Age-CpGs in PWB. Set 1 to 3 were extracted from the same data series (GSE41037) form GEO repository [15], which were grouped according to the ‘dataset’ column of the sample characteristics described in the series matrix file, including 193, 105, 96 healthy Dutch PWB samples, respectively. Set 4 included 274 healthy PWB samples from the ovarian cancer data set reported by Teschendorff [25]. Set 5 and 6 included samples in plate 1 and plate 2 of GSE40279 from GEO repository [26]. As samples in GSE40279 were measured on 11 plates, samples in each plate should be analyzed separately in order to avoid the plate effect. Thus, only samples in plate 1 and 2 with the largest sample sizes were analyzed in this study.

Set 7 included DNA methylation profiles for purified leukocyte subtypes [27], including monocytes (*n* = 5), granulocytes (*n* = 4), neutrophils (*n* = 4), B cells (*n* = 5), NK cells (Pan NKR cells, CD16^+^ NK cells, CD16^−^ NK cells, CD8^+^ NK cells and CD8^−^ NK cells, *n* = 12) and T cells (CD4^+^ T cells, CD8^+^ T cells, NKT cells, Pan T cells and Tregs, *n* = 16). This set was used to identify differentially methylated CpG sites between myeloid cells and lymphoid cells and used to serve as reference to estimate the proportion of leukocyte subtypes in PWB with deconvolution analysis.

Set 8 and Set 9 were used for the detection of Age-CpGs in peripheral CD4^+^ T cells and CD14^+^ monocytes, respectively. The samples included in these two sets were gathered from the Multi-Ethnic study of Atherosclerosis (MESA) which was designed to investigate the prevalence, correlates, and progression of subclinical cardiovascular disease [13]. Set 8 and Set 9 include 214 and 1202 samples, respectively.

Set 10 to 13 were used for the detection of Age-CpGs in brain tissue. The samples in these four sets were consisted of tissue samples of cerebellum, frontal cortex, pons and temporal cortex obtained from 150 neurologically normal Caucasian subjects with no clinical history of neurological or cerebrovascular diseases, respectively [28].

Set 14 to 18 were used to identify AD-CpGs from PWB and brain tissue. These DNAm profiles were determined from 117 normal individuals archived in MRC London Neurodegenerative Disease Brain Bank [5]. The PWB samples of AD patients and normal control (Set 14) were obtained from 57 individuals. The brain tissues of AD patients in Set 15 to 18 included entorhinal cortex, superior temporal gyrus, prefrontal cortex, and cerebellum, respectively.

### Pre-processing DNA methylation data

All of the data sets described in Table 1 were generated using either the Illumina Infinium Human Methylation27k or Methylated450k BeadChip (San Diego, CA, USA). The former platform measures 27, 578 CpG sites within the proximal promoter regions of the transcription start sites of 14, 475 genes, among which 25, 978 sites are also measured on the HumanMethylated450k BeadChip. We focused on analysed the 24, 992 CpG sites commonly measured by the two platforms after removing 1, 486 CpG sites located in X or Y chromosome.

The methylation level of each CpG site was calculated by $\beta =\frac{\max(M,0)}{M+U+100}$ [29], where *M* and *U* represent the methylated and unmethylated signal intensity of this site reported by BeadChip, respectively. Thus, *β* value ranges from 0 (completely unmethylated) to 1 (completely methylated).

To control the quality of each data set, if the missing value rate of a sample was larger than 10%, then this sample was removed; otherwise, the missing value was replaced using the *k*-Nearest neighbour algorithm (*k* = 1). For data sets used to identify Age-CpGs (Set 1 to 13), outlier profiles were removed according to a procedure similar to that described in [30]. Briefly, for a data set, we calculated the inter array correlation between samples across all probes using Pearson correlation, then computed the average of the inter array correlation with other samples calculated for each sample, and finally removed the samples that lie more than two standard deviations from the mean of the average correlations. This procedure was repeated three times. The numbers of removed samples are described in Table 2.

The original platform annotation file was downloaded from GEO (http://www.ncbi.nlm.nih.gov/geo/query/acc.cgi?acc = GPL8490) for Illumina Infinium Human Methylation27 BeadChip and the “Gene_ID” column in this file was used to map each Illumina CpG probe ID to gene ID. Totally, the 24, 992 CpG sites analyzed in this study were mapped to 13, 554 genes.

### Identification of Age-CpGs

To evaluate the associations between age and DNA methylation levels of CpG sites, we fit separate linear regression models with age as a predictor of β value for CpG sites [13]. The covariates were based on the phenotypic traits described in sample characteristic(s) provided by GEO for each data set. For example, the characteristics of samples in Set 8 and Set 9 included sex and race/ethnicity, study site, and residual sample contamination with non-targeted cells. They were used as covariates in linear regression models to find Age-CpGs in CD4^+^ T cells and CD14^+^ monocytes, respectively. P-values were adjusted for multiple testing with the Benjamini-Hochberg procedure to control the FDR at a given level. If a CpG site had significant (FDR adjusted *P*-value < 0.05) positive or negative correlation with age, then it was referred to as an Age-CpG.

To obtain a reliable list of Age-CpGs for PWB, we integrated the Age-CpGs identified from different PWB data sets (Set 1- Set 6) according to the following criteria: significant in at least one data set with an FDR adjusted *P*-value < 0.05 and tentatively significant in at least another data set with an FDR unadjusted P-value < 0.05, after deleting those CpG sites having inconsistent positive or negative correlations with age in any two data sets.

### Identification of AD-CpG sites

To identify AD-CpGs in AD patients compared to normal controls in Set 14 to 18, we employed RankProd method to detect biologically relevant changes [18]. P-values were adjusted for multiple testing using the Benjamini-Hochberg procedure to control the FDR at 0.05.

### Estimation of the proportions of myeloid and lymphoid cells in PWB

Based on the DNAm profiles for purified leukocyte subtypes in Set 7, we quantified the proportion of each leukocyte subtype in each of the PWB sample of Set 1 to 4 by a process of deconvolution proposed by Houseman [16]. Here we did not perform the deconvolution process for Set 5 and 6 as they were produced by the 450K platform, different from the platform (27K) of Set 7 (Table 1). The 500 CpG loci with the most varied methylation levels among these leukocyte subtypes were used as marker loci. If *B* represents the DNAm profile of a PWB sample, *X* represents the proportions of leukocyte subtypes and *A* represents the DNAm profiles for leukocyte subtypes, then

AX~B

The deconvolution is to find the solution of the convolution equation, which will give the cell-type proportions. The proportion of myeloid cells is the accumulated proportion of each cell type coming from myeloid progenitor including monocytes and granulocytes. Similarly, the proportion of lymphoid cells is the accumulated proportion of each cell type coming from lymphoid progenitor including B cells, NK cells and T cells. The detailed algorithm is described in [16].

### Comparison of two lists of CpG sites

For two CpG site lists, if they shared *k* sites, among which *s* were considered as concordant sites, then the concordance rate was calculated as *s*/*k.* The concordant sites were determined as described below:

(1) When comparing two lists of Age-CpGs, a site was considered as a concordant site if it showed the same positive or negative correlations with age in the two lists.

(2) When comparing a list of Age-CpGs to a list of differentially methylated CpG sites between myeloid and lymphoid cells, a site was considered as a concordant site if it was positively (or negatively) correlated with age and correspondingly differentially hypermethylated (or hypomethylated) in myeloid cells compared to lymphoid cells.

(3) When comparing a list of AD-CpGs to a list of differentially methylated CpG sites between myeloid and lymphoid cells, a site was considered as a concordant site if it showed the same hypermethylation or hypomethylation in the two lists.

(4) When comparing a list of AD-CpGs to a list of Age-CpGs, a site was considered as a concordant site if it was differentially hypermethylated (or hypomethylated) between AD patients and normal controls and correspondingly positively (or negatively) correlated with age.

The probability of observing a concordance rate of *s*/*k* by chance was evaluated by the cumulative binomial distribution model as follows:

$$p=1-\sum_{i=0}^{s-1}\left(\begin{array}{c} k\\ i \end{array}\right){\left(p_{e}\right)}^{i}{\left(1-p_{e}\right)}^{k-1}$$

where *P_e_* is the probability of one site having the concordant relationship between the two lists of sites by chance (here, *p*_e_ = 0.5).

### Gene ontology enrichment analysis

Based on the Gene Ontology (GO) database [31], we performed the functional enrichment analysis for the common CpG sites between Age-CpGs and AD-CpGs. The common CpG sites were first mapped to genes. Then, significant GO terms were determined by evaluating whether the ratio of common CpG sites observed in each GO term is significantly larger than that in the background, respectively. The common CpG sites between Age-CpGs and AD-CpGs was considered to be significantly associated with a GO term if the Fisher's exact *P*-value < 0.05.

## SUPPLEMENTARY MATERIAL TABLES



## Abbreviations

AD: Alzheimer's disease
PWB: peripheral whole blood
DNAm: DNA methylation
Age-CpGs: aging related DNAm CpGs
AD-CpGs: Alzheimer's disease related DNAm CpGs
ML-CpGs: differentially methylated CpGs between myeloid cells and lymphoid cells

## References

[1] Cacabelos R, Fernandez-Novoa L, Lombardi V, Kubota Y, Takeda M (2005). Molecular genetics of Alzheimer's disease and aging. Methods Find Exp Clin Pharmacol.

[2] Saito T, Iwata N, Tsubuki S, Takaki Y, Takano J, Huang SM, Suemoto T, Higuchi M, Saido TC (2005). Somatostatin regulates brain amyloid beta peptide Abeta42 through modulation of proteolytic degradation. Nat Med.

[3] Irier HA, Jin P (2012). Dynamics of DNA methylation in aging and Alzheimer's disease. DNA Cell Biol.

[4] Groen TV (2010). DNA Methylation and Alzheimer's Disease. Epigenetics Aging.

[5] Lunnon K, Smith R, Hannon E, De Jager PL, Srivastava G, Volta M, Troakes C, Al-Sarraj S, Burrage J, Macdonald R, Condliffe D, Harries LW, Katsel P, Haroutunian V, Kaminsky Z, Joachim C (2014). Methylomic profiling implicates cortical deregulation of ANK1 in Alzheimer's disease. Nat Neurosci.

[6] Silva PN, Furuya TK, Braga IL, Rasmussen LT, Labio RW, Bertolucci PH, Chen ES, Turecki G, Mechawar N, Payao SL, Mill J, Smith MC (2014). Analysis of HSPA8 and HSPA9 mRNA expression and promoter methylation in the brain and blood of Alzheimer's disease patients. J Alzheimers Dis.

[7] Li L, Choi JY, Lee KM, Sung H, Park SK, Oze I, Pan KF, You WC, Chen YX, Fang JY, Matsuo K, Kim WH, Yuasa Y, Kang D (2012). DNA methylation in peripheral blood: a potential biomarker for cancer molecular epidemiology. J Epidemiol.

[8] Weidner CI, Lin Q, Koch CM, Eisele L, Beier F, Ziegler P, Bauerschlag DO, Jockel KH, Erbel R, Muhleisen TW, Zenke M, Brummendorf TH, Wagner W (2014). Aging of blood can be tracked by DNA methylation changes at just three CpG sites. Genome Biol.

[9] Rakyan VK, Down TA, Maslau S, Andrew T, Yang TP, Beyan H, Whittaker P, McCann OT, Finer S, Valdes AM, Leslie RD, Deloukas P, Spector TD (2010). Human aging-associated DNA hypermethylation occurs preferentially at bivalent chromatin domains. Genome Res.

[10] Alisch RS, Barwick BG, Chopra P, Myrick LK, Satten GA, Conneely KN, Warren ST (2012). Age-associated DNA methylation in pediatric populations. Genome Res.

[11] Reinius LE, Acevedo N, Joerink M, Pershagen G, Dahlen SE, Greco D, Soderhall C, Scheynius A, Kere J (2012). Differential DNA methylation in purified human blood cells: implications for cell lineage and studies on disease susceptibility. PLoS One.

[12] Jaffe AE, Irizarry RA (2014). Accounting for cellular heterogeneity is critical in epigenome-wide association studies. Genome Biol.

[13] Reynolds LM, Taylor JR, Ding J, Lohman K, Johnson C, Siscovick D, Burke G, Post W, Shea S, Jacobs DR, Stunnenberg H, Kritchevsky SB, Hoeschele I, McCall CE, Herrington DM, Tracy RP (2014). Age-related variations in the methylome associated with gene expression in human monocytes and T cells. Nat Commun.

[14] Shad KF, Aghazadeh Y, Ahmad S, Kress B (2013). Peripheral markers of Alzheimer's disease: surveillance of white blood cells. Synapse.

[15] Horvath S, Zhang Y, Langfelder P, Kahn RS, Boks MP, van Eijk K, van den Berg LH, Ophoff RA (2012). Aging effects on DNA methylation modules in human brain and blood tissue. Genome Biol.

[16] Houseman EA, Accomando WP, Koestler DC, Christensen BC, Marsit CJ, Nelson HH, Wiencke JK, Kelsey KT (2012). DNA methylation arrays as surrogate measures of cell mixture distribution. BMC Bioinformatics.

[17] Li H, Zheng T, Chen B, Hong G, Zhang W, Shi T, Li S, Ao L, Wang C, Guo Z (2014). Similar blood-borne DNA methylation alterations in cancer and inflammatory diseases determined by subpopulation shifts in peripheral leukocytes. Br J Cancer.

[18] Yang TY (2015). A Simple Rank Product Approach for Analyzing Two Classes. Bioinform Biol Insights.

[19] Esteve P, Sandonis A, Cardozo M, Malapeira J, Ibanez C, Crespo I, Marcos S, Gonzalez-Garcia S, Toribio ML, Arribas J, Shimono A, Guerrero I, Bovolenta P (2011). SFRPs act as negative modulators of ADAM10 to regulate retinal neurogenesis. Nat Neurosci.

[20] Bertram L, McQueen MB, Mullin K, Blacker D, Tanzi RE (2007). Systematic meta-analyses of Alzheimer disease genetic association studies: the AlzGene database. Nat Genet.

[21] Xu Z, Taylor JA (2014). Genome-wide age-related DNA methylation changes in blood and other tissues relate to histone modification, expression and cancer. Carcinogenesis.

[22] Liu Y, Aryee MJ, Padyukov L, Fallin MD, Hesselberg E, Runarsson A, Reinius L, Acevedo N, Taub M, Ronninger M, Shchetynsky K, Scheynius A, Kere J, Alfredsson L, Klareskog L, Ekstrom TJ (2013). Epigenome-wide association data implicate DNA methylation as an intermediary of genetic risk in rheumatoid arthritis. Nat Biotechnol.

[23] Kaut O, Ramirez A, Pieper H, Schmitt I, Jessen F, Wullner U (2014). DNA methylation of the TNF-alpha promoter region in peripheral blood monocytes and the cortex of human Alzheimer's disease patients. Dement Geriatr Cogn Disord.

[24] Barrett T, Troup DB, Wilhite SE, Ledoux P, Rudnev D, Evangelista C, Kim IF, Soboleva A, Tomashevsky M, Marshall KA, Phillippy KH, Sherman PM, Muertter RN, Edgar R (2009). NCBI GEO: archive for high-throughput functional genomic data. Nucleic Acids Res.

[25] Teschendorff AE, Menon U, Gentry-Maharaj A, Ramus SJ, Gayther SA, Apostolidou S, Jones A, Lechner M, Beck S, Jacobs IJ, Widschwendter M (2009). An epigenetic signature in peripheral blood predicts active ovarian cancer. PLoS One.

[26] Hannum G, Guinney J, Zhao L, Zhang L, Hughes G, Sadda S, Klotzle B, Bibikova M, Fan JB, Gao Y, Deconde R, Chen M, Rajapakse I, Friend S, Ideker T, Zhang K (2013). Genome-wide methylation profiles reveal quantitative views of human aging rates. Mol Cell.

[27] Accomando WP, Wiencke JK, Houseman EA, Butler RA, Zheng S, Nelson HH, Kelsey KT (2012). Decreased NK cells in patients with head and neck cancer determined in archival DNA. Clin Cancer Res.

[28] Gibbs JR, van der Brug MP, Hernandez DG, Traynor BJ, Nalls MA, Lai SL, Arepalli S, Dillman A, Rafferty IP, Troncoso J, Johnson R, Zielke HR, Ferrucci L, Longo DL, Cookson MR, Singleton AB (2010). Abundant quantitative trait loci exist for DNA methylation and gene expression in human brain. PLoS Genet.

[29] Bibikova M, Lin Z, Zhou L, Chudin E, Garcia EW, Wu B, Doucet D, Thomas NJ, Wang Y, Vollmer E, Goldmann T, Seifart C, Jiang W, Barker DL, Chee MS, Floros J (2006). High-throughput DNA methylation profiling using universal bead arrays. Genome Res.

[30] Iancu OD, Darakjian P, Walter NA, Malmanger B, Oberbeck D, Belknap J, McWeeney S, Hitzemann R (2010). Genetic diversity and striatal gene networks: focus on the heterogeneous stock-collaborative cross (HS-CC) mouse. BMC Genomics.

[31] Ashburner M, Ball CA, Blake JA, Botstein D, Butler H, Cherry JM, Davis AP, Dolinski K, Dwight SS, Eppig JT, Harris MA, Hill DP, Issel-Tarver L, Kasarskis A, Lewis S, Matese JC (2000). Gene ontology: tool for the unification of biology. The Gene Ontology Consortium. Nat Genet.

