# Supplementary material for: Common DNA methylation alterations of Alzheimer's disease and aging in peripheral whole blood
Source: Oncotarget. 2016 Mar 2;7(15):19089–98. doi: 10.18632/oncotarget.7862 (PMC4991367; doi:10.18632/oncotarget.7862)
Supplement: Supplementary file 1 [file oncotarget-07-19089-s001.pdf]

Common DNA methylation alterations of Alzheimer's disease and aging in peripheral whole blood

Supplementary Material

Table S1: Correlation of PWB Age-AD-CpGs with age in $CD4^+$ T cells, $CD14^+$ monocytes and four regions of brain tissue

Probe ID	Gene Symbol	Gene ID	PWB		$CD4^+$ T cells		$CD14^+$ monocytes		cerebellum		frontal cortex		pons		temporal cortex	
			state*	P-value	state	P-value	state	P-value	state	P-value	state	P-value	state	P-value	state	P-value
cg01366419	WBSCR17	64409	+	0.0000	+	0.0001	+	0.0901	-	0.0330	+	0.3566	+	0.0305	+	0.0001
cg01410472	CRISPLD1	83690	+	0.0000	+	0.0000	+	0.5439	+	0.0197	+	0.0000	+	0.0000	+	0.0000
cg01589580	CTDP1	9150	-	0.0001	-	0.0255	-	0.2415	-	0.0278	+	0.2164	+	0.3333	+	0.3410
cg01615704	MALL	7851	+	0.0000	+	0.0711	+	0.0000	+	0.0000	+	0.0000	+	0.0000	+	0.0000
cg02388150	SFRP1	6422	+	0.0008	+	0.0031	+	0.0000	+	0.0926	+	0.0000	+	0.0019	+	0.0000
cg02497700	ZNF238	10472	+	0.0012	+	0.0793	+	0.0064	-	0.0130	+	0.4603	-	0.0100	-	0.6713
cg03238797	ADAMTS18	170692	+	0.0002	+	0.0002	+	0.0000	-	0.7887	+	0.0108	+	0.0093	+	0.0016
cg03544320	CRMP1	1400	+	0.0000	+	0.0001	+	0.0000	-	0.1491	+	0.0002	+	0.0018	+	0.0001
cg03775422	MGC33530	222008	+	0.0003	+	0.0015	+	0.0000	+	0.4523	+	0.0002	+	0.0009	+	0.0000
cg03963198	IRX4	50805	+	0.0000	+	0.3126	+	0.0000	+	0.3131	+	0.0000	+	0.0000	+	0.0000
cg04330449	NEUROG1	4762	+	0.0013	+	0.0139	+	0.0001	-	0.2314	+	0.0000	+	0.0029	+	0.0000
cg04457979	KCNQ1DN	55539	+	0.0017	+	0.1487	+	0.0832	-	0.5283	+	0.7617	-	0.3512	-	0.9312
cg04600618	C6orf206	221421	+	0.0004	+	0.1757	+	0.2887	-	0.7296	+	0.0000	+	0.0000	+	0.0000
cg05839235	NPR3	4883	+	0.0000	+	0.0032	+	0.0209	+	0.2193	+	0.0000	+	0.0000	+	0.0000
cg06713098	IGFBP3	3486	+	0.0001	+	0.0110	+	0.0002	+	0.0915	+	0.0065	+	0.0938	+	0.0001
cg06744574	C1orf165	79656	+	0.0003	+	0.7450	+	0.7592	+	0.5837	+	0.4066	+	0.1579	+	0.0000
cg07028533	CNTNAP2	26047	+	0.0001	+	0.0046	+	0.2779	-	0.4541	+	0.0021	+	0.0022	+	0.0001
cg07442479	GDNF	2668	+	0.0000	+	0.1993	+	0.0000	-	0.2232	+	0.0000	+	0.0000	+	0.0000

cg07533148	TRIM58	25893	+	0.0000	+	0.0990	+	0.0466	-	0.5083	+	0.0000	+	0.0000	+	0.0000
cg07846220	LAMA1	284217	+	0.0000	+	0.0000	+	0.1879	+	0.4071	+	0.0005	+	0.0000	+	0.0000
cg07850604	INSM2	84684	+	0.0065	+	0.0000	+	0.0000	+	0.6903	+	0.0000	+	0.0000	+	0.0000
cg08186362	HRH3	11255	+	0.0000	+	0.0000	+	0.0000	-	0.7122	+	0.0000	+	0.0000	+	0.0000
cg08422599	KCNIP1	30820	+	0.0000	+	0.0065	+	0.1041	-	0.5662	+	0.0393	+	0.0008	+	0.0000
cg08441170	MYO3A	53904	+	0.0004	+	0.0072	+	0.0003	-	0.5907	+	0.0000	+	0.0000	+	0.0006
cg08572611	ACTL6B	51412	+	0.0000	+	0.0000	+	0.0000	-	0.6972	+	0.0000	+	0.0000	+	0.0000
cg08575537	EPO	2056	+	0.0000	+	0.0006	+	0.0000	+	0.0063	+	0.0000	+	0.0000	+	0.0000
cg08900043	HOXC10	3226	+	0.0001	+	0.0002	+	0.0000	+	0.0000	+	0.0000	+	0.0000	+	0.0000
cg09053680	UTF1	8433	+	0.0000	+	0.0118	+	0.0352	-	0.1477	+	0.0004	+	0.0000	+	0.0000
cg09156233	BMPR1B	658	+	0.0014	+	0.2166	+	0.1114	-	0.4090	+	0.0003	+	0.0008	+	0.0000
cg09537031	SH2D4A	63898	+	0.0001	+	0.0249	+	0.8544	-	0.8469	+	0.1141	+	0.1281	+	0.0004
cg09601629	FLJ40365	126402	+	0.0029	+	0.2299	+	0.0093	+	0.0628	+	0.0000	+	0.0016	+	0.0003
cg09805010	THRB	7068	+	0.0000	+	0.0006	+	0.9125	-	0.0664	+	0.1888	+	0.0657	+	0.0014
cg10300684	FOXG1B	2290	+	0.0000	+	0.0025	+	0.0037	+	0.0140	+	0.0000	+	0.0000	+	0.0000
cg10303487	DPYS	1807	+	0.0000	+	0.0021	+	0.0000	-	0.4822	+	0.0000	+	0.0000	+	0.0000
cg10313673	CILP2	148113	+	0.0000	+	0.0320	+	0.0000	+	0.5447	+	0.3206	-	0.0149	+	0.0016
cg10708905	LRIG3	121227	+	0.0002	+	0.0062	+	0.3058	-	0.6226	+	0.3852	+	0.3075	+	0.0050
cg11011938	SEMA5A	9037	+	0.0000	+	0.0012	+	0.0000	+	0.0000	+	0.0000	+	0.0000	+	0.0000
cg11051843	SEMA3C	10512	+	0.0000	+	0.0066	-	0.2643	+	0.1192	+	0.1131	+	0.0080	+	0.0000
cg11428724	PAX7	5081	+	0.0000	+	0.0026	+	0.0106	-	0.2785	+	0.0000	+	0.0216	+	0.0000
cg11884546	ITGAX	3687	-	0.0019	+	0.1129	-	0.2725	+	0.0000	+	0.0199	+	0.1555	+	0.0060
cg12277666	TDRD5	163589	+	0.0000	+	0.0016	+	0.0000	+	0.0287	+	0.0002	+	0.0000	+	0.0000
cg12497564	RBP1	5947	+	0.0000	+	0.0003	+	0.0001	+	0.9191	+	0.0000	+	0.0005	+	0.0000
cg13265789	UNC5C	8633	+	0.0004	+	0.0015	-	0.5498	+	0.8951	-	0.5170	+	0.9926	+	0.0890

cg13359415	LGI2	55203	+	0.0022	+	0.0212	+	0.2622	-	0.1485	+	0.1255	+	0.0011	+	0.0002
cg14155416	L3MBTL4	91133	+	0.0000	+	0.0045	+	0.0846	+	0.3226	+	0.0008	+	0.0008	+	0.0000
cg14603345	BTBD3	22903	+	0.0000	+	0.0000	+	0.0000	-	0.5192	+	0.6524	+	0.0037	+	0.0224
cg15250507	C1orf106	55765	+	0.0000	+	0.0100	+	0.1451	-	0.0991	+	0.5767	-	0.4821	+	0.1603
cg15319457	HES6	55502	+	0.0000	+	0.0001	+	0.0000	+	0.0001	+	0.0000	+	0.0000	+	0.0000
cg15543551	FGF12	2257	+	0.0003	-	0.7495	+	0.0329	-	0.2995	+	0.4711	+	0.1643	+	0.0818
cg15731815	C1orf188	148646	+	0.0000	+	0.0018	+	0.0032	+	0.0000	+	0.0000	+	0.0000	+	0.0000
cg16041660	PRICKLE1	144165	+	0.0001	+	0.0015	-	0.9466	+	0.7300	+	0.0000	+	0.0000	+	0.0000
cg16773899	EDIL3	10085	+	0.0000	+	0.0002	+	0.4167	-	0.3457	-	0.8476	-	0.4354	+	0.6466
cg16924616	DLX5	1749	+	0.0001	+	0.0003	+	0.0000	-	0.5600	+	0.0000	+	0.0000	+	0.0000
cg17272843	KCTD14	65987	+	0.0011	+	0.2732	+	0.1365	-	0.7067	+	0.0054	+	0.0186	+	0.0065
cg17457560	NRG1	3084	+	0.0000	+	0.0044	+	0.0000	+	0.4949	+	0.0000	+	0.0000	+	0.0000
cg17733100	C1orf106	55765	+	0.0000	+	0.0054	+	0.2339	+	0.0761	+	0.0041	+	0.4481	+	0.0000
cg17977409	SH3GL2	6456	+	0.0000	+	0.0000	+	0.0248	-	0.4743	+	0.0567	+	0.0000	+	0.0000
cg18182399	DES	1674	+	0.0000	+	0.0036	+	0.0000	+	0.0000	+	0.0000	+	0.0000	+	0.0000
cg18239753	KHDRBS2	202559	+	0.0000	+	0.0002	+	0.0912	+	0.9844	+	0.6871	+	0.1063	+	0.3009
cg18503260	CA13	377677	+	0.0000	+	0.2322	-	0.1693	-	0.0810	+	0.0000	+	0.0000	+	0.0000
cg18815943	FOXE3	2301	+	0.0000	+	0.0000	+	0.0000	+	0.0217	+	0.0000	+	0.0000	+	0.0000
cg20052718	TWIST1	7291	+	0.0000	+	0.0001	+	0.0807	+	0.0003	+	0.0000	+	0.0000	+	0.0000
cg20098887	RAB32	10981	+	0.0000	+	0.0067	+	0.6223	+	0.0428	+	0.0000	+	0.0000	+	0.0000
cg20616414	WNK2	65268	+	0.0001	+	0.4155	+	0.0000	-	0.0307	+	0.0000	+	0.0000	+	0.0000
cg20678353	FLJ35695	400359	-	0.0010	+	0.5626	-	0.0436	-	0.7812	-	0.4236	-	0.0095	+	0.9746
cg20723355	FBXO39	162517	+	0.0000	+	0.0002	+	0.0000	-	0.8097	+	0.0000	+	0.0000	+	0.0000
cg20937139	PDGFC	56034	+	0.0000	+	0.0006	+	0.0086	+	0.8840	+	0.1049	+	0.0151	+	0.0168
cg21172540	TSSK3	81629	+	0.0000	+	0.0015	+	0.0162	-	0.0809	+	0.0000	+	0.0916	+	0.0000

cg21184011	NHN1	124245	-	0.0005	-	0.2513	-	0.0000	-	0.3704	-	0.7173	-	0.4864	-	0.0395
cg21238818	GAL3ST3	89792	+	0.0000	+	0.0023	+	0.0025	-	0.7542	+	0.5747	+	0.2072	-	0.6165
cg21269934	FLJ37478	339983	+	0.0000	+	0.0247	+	0.2264	-	0.1101	+	0.0221	+	0.0000	+	0.0138
cg21494776	ICAM4	3386	+	0.0000	+	0.0017	+	0.0197	+	0.1289	+	0.0011	+	0.2015	+	0.0000
cg21790626	ZNF154	7710	+	0.0013	+	0.0086	+	0.0000	+	0.3828	+	0.0106	+	0.0000	+	0.0034
cg21902544	CBLN2	147381	+	0.0000	+	0.4362	+	0.6749	+	0.0391	+	0.0276	+	0.2618	+	0.1139
cg22375192	IGF1R	3480	+	0.0021	+	0.2360	-	0.7212	+	0.3296	+	0.4665	-	0.8440	+	0.0126
cg22380033	GRB14	2888	+	0.0000	+	0.0008	+	0.5732	-	0.0024	+	0.1065	+	0.1411	+	0.0759
cg22598028	ZNF660	285349	+	0.0004	+	0.0001	+	0.4395	-	0.2526	+	0.1716	+	0.0020	+	0.3548
cg22660578	LHX1	3975	+	0.0000	+	0.3216	+	0.0000	+	0.7959	+	0.0000	+	0.0000	+	0.0000
cg22879515	BTG4	54766	+	0.0012	+	0.5685	-	0.8714	+	0.5857	+	0.1086	+	0.0693	+	0.0270
cg23290344	NEF3	4741	+	0.0000	+	0.0000	+	0.0000	-	0.3807	+	0.0000	+	0.0000	+	0.0000
cg23349790	IGSF21	84966	+	0.0013	+	0.1697	+	0.1277	-	0.6935	+	0.6814	-	0.7642	+	0.0082
cg23695504	FLJ45717	388759	+	0.0000	+	0.0001	+	0.0009	-	0.4073	+	0.0110	+	0.2710	+	0.0000
cg23850212	ZFP28	140612	+	0.0001	+	0.0151	-	0.4842	-	0.5876	+	0.0309	+	0.0003	+	0.0000
cg24120841	THRB	7068	+	0.0000	+	0.0001	+	0.8982	-	0.2109	+	0.0907	+	0.0020	+	0.0034
cg24826867	IRF8	3394	+	0.0000	-	0.6925	+	0.1790	-	0.4228	+	0.0000	+	0.0000	+	0.0000
cg24891133	FLJ14834	84935	+	0.0000	+	0.0275	+	0.0000	+	0.7591	+	0.0007	+	0.0000	+	0.0000
cg25437385	SLC35F3	148641	+	0.0001	+	0.0000	+	0.0379	-	0.1713	+	0.7540	+	0.2042	+	0.6889
cg25511429	NRN1	51299	+	0.0002	+	0.4914	+	0.0000	+	0.0004	+	0.0000	+	0.0000	+	0.0000
cg25691167	FERD3L	222894	+	0.0017	+	0.0000	+	0.0000	-	0.0225	-	0.0933	+	0.5641	-	0.7474
cg25784308	FLJ38377	205147	+	0.0003	+	0.0010	+	0.0000	+	0.4274	+	0.0000	+	0.0000	+	0.0000
cg25971347	FOXF1	2294	+	0.0005	+	0.3107	-	0.3369	+	0.9488	+	0.0000	+	0.0223	+	0.0000
cg26024843	COL5A1	1289	+	0.0000	+	0.0806	-	0.0235	+	0.0983	+	0.0000	+	0.0000	+	0.0000
cg26186727	NETO1	81832	+	0.0000	+	0.0004	+	0.0429	+	0.6789	+	0.0102	+	0.0000	+	0.1085

cg26252167	GPR6	2830	+	0.0000	+	0.0019	+	0.0000	-	0.8561	+	0.0025	+	0.0000	+	0.0003
cg26521404	HOXA9	3205	+	0.0016	+	0.0489	+	0.0246	+	0.7866	+	0.0000	+	0.0000	+	0.0004
cg27009703	HOXA9	3205	+	0.0000	+	0.0002	+	0.0331	+	0.0002	+	0.0000	+	0.0000	+	0.0000
cg27268486	GNAI1	2770	+	0.0004	+	0.0000	+	0.6662	-	0.7932	+	0.0679	+	0.0778	+	0.0000

*: + indicates positive correlation; - indicates negative correlation.

Table S2: GO terms enriched by common AD-CpGs and Age-CpGs in PWB

GO ID	GO annotation	P-value	GO ID	GO annotation	P-value
GO:0048856	anatomical structure development	0.0000	GO:0042127	regulation of cell proliferation	0.0123
GO:0048731	system development	0.0000	GO:0048812	neuron projection morphogenesis	0.0123
GO:0007275	multicellular organismal development	0.0000	GO:0048667	cell morphogenesis involved in neuron differentiation	0.0123
GO:0007399	nervous system development	0.0000	GO:0001501	skeletal system development	0.0123
GO:0032502	developmental process	0.0000	GO:2000112	regulation of cellular macromolecule biosynthetic process	0.0123
GO:0048869	cellular developmental process	0.0000	GO:0007411	axon guidance	0.0123
GO:0030154	cell differentiation	0.0000	GO:0014033	neural crest cell differentiation	0.0123
GO:0048513	organ development	0.0000	GO:0032989	cellular component morphogenesis	0.0134
GO:0032501	multicellular organismal process	0.0000	GO:0050789	regulation of biological process	0.0139
GO:0048699	generation of neurons	0.0000	GO:0007417	central nervous system development	0.0153
GO:0009790	embryo development	0.0001	GO:0050793	regulation of developmental process	0.0163
GO:0030182	neuron differentiation	0.0001	GO:0010556	regulation of macromolecule biosynthetic process	0.0168
GO:0022008	neurogenesis	0.0001	GO:0016477	cell migration	0.0168
GO:0009887	organ morphogenesis	0.0001	GO:0045449	regulation of transcription	0.0170
GO:0009653	anatomical structure morphogenesis	0.0002	GO:0048666	neuron development	0.0224
GO:0009888	tissue development	0.0008	GO:0080090	regulation of primary metabolic process	0.0251
GO:0048468	cell development	0.0013	GO:0006928	cellular component movement	0.0257
GO:0040011	locomotion	0.0020	GO:0048870	cell motility	0.0257
GO:0008284	positive regulation of cell proliferation	0.0025	GO:0051674	localization of cell	0.0257
GO:0048598	embryonic morphogenesis	0.0032	GO:0045935	positive regulation of nucleobase, nucleoside, nucleotide and nucleic acid metabolic process	0.0285
GO:0003002	regionalization	0.0044	GO:0031175	neuron projection development	0.0293

GO:0019219	regulation of nucleobase, nucleoside, nucleotide and nucleic acid metabolic process	0.0046	GO:0031323	regulation of cellular metabolic process	0.0293
GO:0031326	regulation of cellular biosynthetic process	0.0049	GO:2000026	regulation of multicellular organismal development	0.0297
GO:0007389	pattern specification process	0.0056	GO:0006355	regulation of transcription, DNA-dependent	0.0320
GO:0009889	regulation of biosynthetic process	0.0057	GO:0010557	positive regulation of macromolecule biosynthetic process	0.0340
GO:0051171	regulation of nitrogen compound metabolic process	0.0073	GO:0051173	positive regulation of nitrogen compound metabolic process	0.0340
GO:0035107	appendage morphogenesis	0.0080	GO:0045198	establishment of epithelial cell apical/basal polarity	0.0367
GO:0035108	limb morphogenesis	0.0080	GO:0065007	biological regulation	0.0367
GO:0048858	cell projection morphogenesis	0.0082	GO:0045595	regulation of cell differentiation	0.0379
GO:0007409	axonogenesis	0.0082	GO:0001738	morphogenesis of a polarized epithelium	0.0388
GO:0032990	cell part morphogenesis	0.0084	GO:0030030	cell projection organization	0.0425
GO:0048736	appendage development	0.0092	GO:0051252	regulation of RNA metabolic process	0.0428
GO:0060173	limb development	0.0092	GO:0051239	regulation of multicellular organismal process	0.0457
GO:0048732	gland development	0.0092	GO:0060485	mesenchyme development	0.0480
GO:0000902	cell morphogenesis	0.0103	GO:0060255	regulation of macromolecule metabolic process	0.0485
GO:0009952	anterior/posterior pattern formation	0.0107	GO:0006351	transcription, DNA-dependent	0.0499
GO:0000904	cell morphogenesis involved in differentiation	0.0116	GO:0048729	tissue morphogenesis	0.0499

Table S3: Methylation alterations of PWB Age-AD-CpGs in four regions of brain tissue of AD patients

Probe ID	Gene Symbol	Gene ID	PWB		cerebellum		entorhinal cortex		prefrontal cortex		superior temporal gyrus	
			status*	P-value	status	P-value	status	P-value	status	P-value	status	P-value
cg01366419	WBSCR17	64409	1	0.0431	0	>0.05	0	>0.05	0	>0.05	0	>0.05
cg01410472	CRISPLD1	83690	1	0.04	0	>0.05	0	>0.05	0	>0.05	0	>0.05
cg01589580	CTDP1	9150	-1	0.0004	0	>0.05	0	>0.05	0	>0.05	1	0.0368
cg01615704	MALL	7851	1	0.0077	0	>0.05	0	>0.05	0	>0.05	0	>0.05
cg02388150	SFRP1	6422	1	0.0118	0	>0.05	0	>0.05	1	0.0071	0	>0.05
cg02497700	ZNF238	10472	1	0.0067	0	>0.05	0	>0.05	0	>0.05	0	>0.05
cg03238797	ADAMTS18	170692	1	0.0026	0	>0.05	0	>0.05	1	0	1	0
cg03544320	CRMP1	1400	1	0.0009	0	>0.05	0	>0.05	1	0.0016	1	0.047
cg03775422	MGC33530	222008	1	0.0484	0	>0.05	1	0.0005	0	>0.05	0	>0.05
cg03963198	IRX4	50805	1	0.0026	-1	0.0411	-1	0.0275	0	>0.05	0	>0.05
cg04330449	NEUROG1	4762	1	0.0402	0	>0.05	1	0.0064	0	>0.05	-1	0.0314
cg04457979	KCNQ1DN	55539	1	0.0078	1	0.0201	1	0.022	1	0.016	1	0.004
cg04600618	C6orf206	221421	1	0.0024	0	>0.05	0	>0.05	0	>0.05	1	0.034
cg05839235	NPR3	4883	1	0.0444	0	>0.05	0	>0.05	0	>0.05	0	>0.05
cg06713098	IGFBP3	3486	1	0.002	0	>0.05	0	>0.05	0	>0.05	0	>0.05
cg06744574	C1orf165	79656	1	0.032	0	>0.05	0	>0.05	0	>0.05	0	>0.05
cg07028533	CNTNAP2	26047	1	0.0461	0	>0.05	0	>0.05	0	>0.05	0	>0.05
cg07442479	GDNF	2668	1	0.032	0	>0.05	0	>0.05	1	0.0129	0	>0.05
cg07533148	TRIM58	25893	1	0.0141	0	>0.05	0	>0.05	1	0.0343	0	>0.05
cg07846220	LAMA1	284217	1	0.0012	0	>0.05	0	>0.05	0	>0.05	0	>0.05
cg07850604	INSM2	84684	1	0.0466	0	>0.05	-1	0.0354	0	>0.05	1	0.0032
cg08186362	HRH3	11255	1	0.0013	0	>0.05	0	>0.05	0	>0.05	0	>0.05

cg08422599	KCNIP1	30820	1	0.021	0	>0.05	-1	0.0051	0	>0.05	0	>0.05
cg08441170	MYO3A	53904	1	0.0007	0	>0.05	1	0.0448	0	>0.05	0	>0.05
cg08572611	ACTL6B	51412	1	0.0352	0	>0.05	1	0.0389	0	>0.05	1	0.0092
cg08575537	EPO	2056	1	0.0008	0	>0.05	0	>0.05	0	>0.05	0	>0.05
cg08900043	HOXC10	3226	1	0.0339	0	>0.05	1	0.0472	0	>0.05	0	>0.05
cg09053680	UTF1	8433	1	0.0001	0	>0.05	0	>0.05	0	>0.05	0	>0.05
cg09156233	BMPR1B	658	1	0.0001	0	>0.05	0	>0.05	0	>0.05	0	>0.05
cg09537031	SH2D4A	63898	1	0.0339	1	0	1	0.0447	0	>0.05	0	>0.05
cg09601629	FLJ40365	126402	1	0.0059	0	>0.05	0	>0.05	0	>0.05	0	>0.05
cg09805010	THRB	7068	1	0.0013	0	>0.05	0	>0.05	1	0.0143	1	0.0241
cg10300684	FOXG1B	2290	1	0.0088	0	>0.05	1	0	1	0.0019	-1	0.0347
cg10303487	DPYS	1807	1	0	0	>0.05	-1	0.0464	0	>0.05	0	>0.05
cg10313673	CILP2	148113	1	0.0044	0	>0.05	0	>0.05	0	>0.05	0	>0.05
cg10708905	LRIG3	121227	1	0.019	0	>0.05	0	>0.05	0	>0.05	0	>0.05
cg11011938	SEMA5A	9037	1	0.0388	0	>0.05	0	>0.05	0	>0.05	0	>0.05
cg11051843	SEMA3C	10512	1	0.0239	0	>0.05	0	>0.05	-1	0.0319	0	>0.05
cg11428724	PAX7	5081	1	0.0048	1	0.0004	0	>0.05	1	0.0145	1	0.0145
cg11884546	ITGAX	3687	-1	0.0017	0	>0.05	0	>0.05	0	>0.05	0	>0.05
cg12277666	TDRD5	163589	1	0.0071	0	>0.05	0	>0.05	0	>0.05	0	>0.05
cg12497564	RBP1	5947	1	0.0001	0	>0.05	0	>0.05	0	>0.05	0	>0.05
cg13265789	UNC5C	8633	1	0.0348	0	>0.05	0	>0.05	0	>0.05	0	>0.05
cg13359415	LGI2	55203	1	0.0016	0	>0.05	1	0	1	0	0	>0.05
cg14155416	L3MBTL4	91133	1	0.0019	0	>0.05	0	>0.05	0	>0.05	0	>0.05
cg14603345	BTBD3	22903	1	0.0019	0	>0.05	0	>0.05	1	0.0475	0	>0.05
cg15250507	C1orf106	55765	1	0.0007	0	>0.05	1	0.001	0	>0.05	0	>0.05

cg15319457	HES6	55502	1	0.0007	0	>0.05	0	>0.05	1	0.0004	1	0
cg15543551	FGF12	2257	1	0.0162	0	>0.05	0	>0.05	0	>0.05	0	>0.05
cg15731815	C1orf188	148646	1	0.0021	0	>0.05	0	>0.05	0	>0.05	0	>0.05
cg16041660	PRICKLE1	144165	1	0.0384	1	0.0195	1	0.0362	0	>0.05	0	>0.05
cg16773899	EDIL3	10085	1	0.0046	1	0.0353	0	>0.05	0	>0.05	0	>0.05
cg16924616	DLX5	1749	1	0.0006	0	>0.05	0	>0.05	0	>0.05	1	0.0065
cg17272843	KCTD14	65987	1	0.0222	0	>0.05	0	>0.05	0	>0.05	0	>0.05
cg17457560	NRG1	3084	1	0.041	0	>0.05	0	>0.05	0	>0.05	0	>0.05
cg17733100	C1orf106	55765	1	0.0296	0	>0.05	-1	0.0002	-1	0.0023	0	>0.05
cg17977409	SH3GL2	6456	1	0.0034	0	>0.05	0	>0.05	0	>0.05	0	>0.05
cg18182399	DES	1674	1	0.0141	0	>0.05	0	>0.05	0	>0.05	0	>0.05
cg18239753	KHDRBS2	202559	1	0.0286	0	>0.05	1	0.0047	1	0	1	0.0002
cg18503260	CA13	377677	1	0.0314	0	>0.05	0	>0.05	-1	0.0343	0	>0.05
cg18815943	FOXE3	2301	1	0.0191	0	>0.05	0	>0.05	0	>0.05	0	>0.05
cg20052718	TWIST1	7291	1	0.0445	0	>0.05	-1	0.0009	0	>0.05	0	>0.05
cg20098887	RAB32	10981	1	0.0295	0	>0.05	0	>0.05	0	>0.05	0	>0.05
cg20616414	WNK2	65268	1	0.0401	0	>0.05	0	>0.05	0	>0.05	0	>0.05
cg20678353	FLJ35695	400359	-1	0.036	0	>0.05	0	>0.05	-1	0.0337	0	>0.05
cg20723355	FBXO39	162517	1	0.0295	0	>0.05	0	>0.05	0	>0.05	1	0.0058
cg20937139	PDGFC	56034	1	0.0058	0	>0.05	0	>0.05	0	>0.05	0	>0.05
cg21172540	TSSK3	81629	1	0.0131	0	>0.05	0	>0.05	0	>0.05	0	>0.05
cg21184011	NHN1	124245	-1	0.0025	0	>0.05	0	>0.05	0	>0.05	0	>0.05
cg21238818	GAL3ST3	89792	1	0.032	0	>0.05	0	>0.05	0	>0.05	0	>0.05
cg21269934	FLJ37478	339983	1	0.0237	0	>0.05	0	>0.05	0	>0.05	0	>0.05
cg21494776	ICAM4	3386	1	0.0077	0	>0.05	0	>0.05	0	>0.05	0	>0.05

cg21790626	ZNF154	7710	1	0.0308	0	>0.05	0	>0.05	0	>0.05	1	0.0109
cg21902544	CBLN2	147381	1	0.0088	0	>0.05	0	>0.05	0	>0.05	1	0.0016
cg22375192	IGF1R	3480	1	0.0065	0	>0.05	0	>0.05	0	>0.05	0	>0.05
cg22380033	GRB14	2888	1	0.0173	0	>0.05	1	0.0001	1	0	1	0
cg22598028	ZNF660	285349	1	0.0013	0	>0.05	0	>0.05	0	>0.05	0	>0.05
cg22660578	LHX1	3975	1	0.0012	0	>0.05	0	>0.05	0	>0.05	1	0.0277
cg22879515	BTG4	54766	1	0.0122	0	>0.05	0	>0.05	0	>0.05	0	>0.05
cg23290344	NEF3	4741	1	0.0005	0	>0.05	0	>0.05	0	>0.05	0	>0.05
cg23349790	IGSF21	84966	1	0.0033	0	>0.05	0	>0.05	0	>0.05	0	>0.05
cg23695504	FLJ45717	388759	1	0.0001	-1	0.0134	-1	0.0072	0	>0.05	1	0.0009
cg23850212	ZFP28	140612	1	0.0011	0	>0.05	0	>0.05	1	0.0393	0	>0.05
cg24120841	THRB	7068	1	0.0146	0	>0.05	0	>0.05	0	>0.05	0	>0.05
cg24826867	IRF8	3394	1	0.0013	-1	0.0014	0	>0.05	-1	0.0009	0	>0.05
cg24891133	FLJ14834	84935	1	0.0452	0	>0.05	0	>0.05	0	>0.05	0	>0.05
cg25437385	SLC35F3	148641	1	0.0447	0	>0.05	0	>0.05	0	>0.05	0	>0.05
cg25511429	NRN1	51299	1	0.0453	0	>0.05	0	>0.05	0	>0.05	0	>0.05
cg25691167	FERD3L	222894	1	0.0443	0	>0.05	0	>0.05	0	>0.05	0	>0.05
cg25784308	FLJ38377	205147	1	0.0118	0	>0.05	1	0	1	0	1	0
cg25971347	FOXF1	2294	1	0.0099	0	>0.05	-1	0.0032	1	0.0005	1	0
cg26024843	COL5A1	1289	1	0.0429	0	>0.05	0	>0.05	0	>0.05	0	>0.05
cg26186727	NETO1	81832	1	0.0467	0	>0.05	0	>0.05	0	>0.05	0	>0.05
cg26252167	GPR6	2830	1	0.0493	-1	0.0346	0	>0.05	0	>0.05	0	>0.05
cg26521404	HOXA9	3205	1	0.0001	0	>0.05	0	>0.05	0	>0.05	0	>0.05
cg27009703	HOXA9	3205	1	0.0007	0	>0.05	0	>0.05	0	>0.05	0	>0.05
cg27268486	GNAI1	2770	1	0.0399	0	>0.05	0	>0.05	0	>0.05	0	>0.05

*: 1 indicates hypermethylated; -1 indicates hypomethylated; 0 indicates unchanged.
